# Physical Interactions and Functional Coordination between the Core Subunits of Set1/Mll Complexes and the Reprogramming Factors

**DOI:** 10.1371/journal.pone.0145336

**Published:** 2015-12-21

**Authors:** Zhenhua Yang, Jonathan Augustin, Jing Hu, Hao Jiang

**Affiliations:** Department of Biochemistry and Molecular Genetics and the UAB Stem Cell Institute, University of Alabama at Birmingham School of Medicine, Birmingham, Alabama, United States of America; University of Wisconsin - Madison, UNITED STATES

## Abstract

Differentiated cells can be reprogrammed to the pluripotent state by overexpression of defined factors, and this process is profoundly influenced by epigenetic mechanisms including dynamic histone modifications. Changes in H3K4 methylation have been shown to be the predominant activating response in the early stage of cellular reprogramming. Mechanisms underlying such epigenetic priming, however, are not well understood. Here we show that the expression of the reprogramming factors (Yamanaka factors, Oct4, Sox2, Klf4 and Myc), especially Myc, directly promotes the expression of certain core subunits of the Set1/Mll family of H3K4 methyltransferase complexes. A dynamic recruitment of the Set1/Mll complexes largely, though not sufficiently in its own, explains the dynamics of the H3K4 methylation during cellular reprogramming. We then demonstrate that the core subunits of the Set1/Mll complexes physically interact with mainly Sox2 and Myc among the Yamanaka factors. We further show that Sox2 directly binds the Ash2l subunit in the Set1/Mll complexes and this binding is mediated by the HMG domain of Sox2. Functionally, we show that the Set1/Mll complex core subunits are required for efficient cellular reprogramming. We also show that Dpy30, one of the core subunits in the complexes, is required for the efficient target binding of the reprogramming factors. Interestingly, such requirement is not necessarily dependent on locus-specific H3K4 methylation. Our work provides a better understanding of how the reprogramming factors physically interact and functionally coordinate with a key group of epigenetic modulators to mediate transitions of the chromatin state involved in cellular reprogramming.

## Introduction

The seminal discovery that differentiated cells can be reprogrammed to induced pluripotent stem cells (iPS cells) by four transcription factors Oct4, Sox2, Klf4, and c-Myc (O, S, K, M, the Yamanaka factors) [1] represents a major conceptual breakthrough in our understanding of the fundamental mechanisms controlling cell identity, and has a huge potential to revolutionize regenerative medicine. However, a number of issues, including inefficient and incomplete reprogramming and tumorigenic risks, need to be resolved before fully realizing the potential of iPS cells [2]. Directed by key transcription factors, cellular reprogramming is accompanied by extensive remodeling of epigenetic marks, and mounting evidence supports the profound influence of epigenetic regulators on reprogramming [3–11]. Moreover, chemicals acting on epigenetic modifications have been shown to be able to functionally replace some of the original transcription factors in reprogramming or enhance the reprogramming efficiency [12–14]. These findings underscore the importance of a deep comprehension of the epigenetic mechanisms for improved reprogramming.

At the very early stage of reprogramming upon OSKM expression, H3K4 methylation was found to be a predominant activating response globally—it is established de novo or significantly enhanced at large subsets of pluripotency-related or developmental gene promoters preceding the loading of the general transcription machinery [15, 16]. These results suggest that the reprogramming factors, rather than RNA polymerase II, directly or indirectly promote the dynamic changes of the histone mark, and thereby initiate a concerted change in the target chromatin environment which may epigenetically prime the subsequent transcription change. Some other pluripotency-associated genes gain promoter H3K4 methylation at the late or final stage of reprogramming [15, 17, 18]. However, the functional significance of the locus-specific H3K4 methylation in reprogramming is less clear. Moreover, it remains incompletely understood how the reprogramming factors elicit the alteration of locus-specific H3K4 methylation, which can be potentially affected by many factors including the local enzyme concentration through regulated recruitment, the enzymatic activities, and the chromatin and histone status.

In mammals, the most notable H3K4 methyltransferases are the Set1/Mll family complexes [19–21]. Apart from some specialized subunits, these complexes contain either Set1a, Set1b, Mll1, Mll2, Mll3, or Mll4 as the catalytic subunit and Wdr5, Rbbp5, Ash2l, and Dpy30 as integral core subunits that are also important for the efficient methylation activity of the complexes [22–27]. Several of these subunits have been linked to either the maintenance or the execution of pluripotency. Wdr5, Ash2l, and Set1a are important for maintenance of an undifferentiated state of mouse embryonic stem (ES) cells [28–30], and Wdr5 and Set1a are also essential for cellular reprogramming to pluripotency [28, 29]. However, Rbbp5 or Dpy30 knockdown (KD) in mouse ES cells does not significantly affect their self-renewal, but prevents their efficient differentiation [27]. Other than the presence of Wdr5 in several complexes different from H3K4 methyltransferases [31–34], reasons for the discrepant effects among these subunits remain unclear. Although depletion of these subunits all affect bulk H3K4 methylation, it is difficult to functionally attribute the cellular effects to the locus-specific methylation.

Mechanisms that control the recruitment of histone modifiers remain a central and largely open question in epigenetics. Several types of mechanisms may be involved in the genomic recruitment of H3K4 methyltransferases [19, 35]. Preexisting H3K4 methylation is likely established by mechanisms intrinsic to DNA sequence including CpG islands which interact with the CXXC domain in some of the H3K4 methyltransferase complex components such as CFP1 [36, 37]. Transcription factors can recruit histone modifiers to genomic sites for histone modifications and transcription [26, 38]. It has not been systematically examined whether the Yamanaka factors, all of which are transcription factors, physically recruit the methylation enzymes to their genomic targets in cellular reprogramming.

In this report, we started with an observation that ectopic expression of the reprogramming factors can promote the expression of certain core subunits of the H3K4 methyltransferase complexes, and characterized an important role of the core subunits of these complexes in cellular reprogramming through physical interactions and functional coordination with the reprograming factors. Our results provide a better understanding of the epigenetic priming and regulation involved in cellular reprogramming toward pluripotency.

## Materials and Methods

### Lentiviruses, cell culture, reprogramming, and gene knockdown

The lentiviral constructs for FUW-M2rtTA (plasmid #20342) [39], TetO-FUW-OSKM (plasmid #20321) [40], TetO-FUW-Oct4 (plasmid #20323) [41], TetO-FUW-Sox2 (plasmid #20326) [41], TetO-FUW-Klf4 (plasmid #20322) [41], and TetO-FUW-Myc (plasmid #20324) [41] were all from Addgene. FLAG-HA-tagged individual mouse O, S, K and M-expressing lentiviral constructs were made based on TetO-FUW-OSKM. Lentiviruses for Rbbp5 and Dpy30 shRNAs and viral particle production were described [27].

HEK293T cells were obtained from American Type Culture Collection (ATCC, CRL-11268), and cultured in DMEM [Gibco] with 10% fetal bovine serum [FBS] [Gibco]. Oct4-GFP mouse embryonic fibroblasts (MEFs) harbor a GFP reporter gene downstream of exon 5 of the endogenous *Oct4* locus [41], and were purchased from Stemgent (Cat# 08–0028, San Diego, CA) at passage 2. They were cultured in MEF culture medium (L-glutamine-containing DMEM with 10% FBS, 0.1 mM non-essential amino acids, and 55μM β-mercaptoethanol, all from Invitrogen), and passage was kept at minimum. For stable KD, MEFs were infected with lentiviruses expressing control, Rbbp5, or Dpy30 shRNA, followed by puromycin (2μg/ml) selection for 2–3 days starting from 2 days after infection. For reprogramming, MEFs were infected with FUW-M2rtTA and TetO-FUW-OSKM lentiviruses, and were cultured on the next day on 0.1% gelatin-coated tissue-culture plates in the reprogramming medium (knockout DMEM [Gibco], 15% ES-certified FBS [Gibco], 2mM L-glutamine, 0.1mM non-essential amino acids, and 0.1mM β-mercaptoethanol, recombinant leukemia inhibitory factor [LIF] [27], 50μg/ml vitamin C [42], and 2μg/ml doxycycline). P493-6 cells [43] were a kind gift from Alanna Ruddell (Fred Hutchinson Cancer Research Center, Seattle) with the permission of Dirk Eick (Helmholtz Center Munich, Germany), and were cultured in RPMI 1640 medium with 10% FBS.

### Protein-protein interaction by co-immunoprecipitation (co-IP)

For co-IP in MEFs, MEFs were infected with indicated lentivirus expressing individual reprogramming factors, followed by induction by doxycycline at 1μg/ml for 48 hours. For co-IP in 293 cells, FLAG-HA-tagged mouse O, S, K and M cDNAs were cloned into pcDNA5/FRT/TO (Invitrogen). Cell lines stably expressing these factors were generated by co-transfection of these individual constructs with pOG44 (Invitrogen) into Flp-In T-REx-293 cells (Invitrogen), followed by hygromycin B selection. Selected clones were induced by doxycycline at 1μg/ml for 48 hours before Co-IP. To map Sox2 binding, indicated truncation mutants were generated in pcDNA5/FRT/TO with FLAG-HA tag, and transiently transfected into 293T cells for co-IP. Cells were harvested and lysed in a buffer containing BC300 (50 mM Tris [pH 7.4], 300 mM KCl, 20% glycerol, 0.2mM EDTA), 0.1% NP40 and 1x protease inhibitor cocktail (Roche). Cleared lysates were diluted by equal volume of BC0 (50 mM Tris [pH 7.4], 20% glycerol, 0.2mM EDTA) and protease inhibitors, so that the final condition was identical to the IP buffer (BC150, 0.05% NP40 and protease inhibitors). Co-IP was carried out by rocking the diluted lysates with anti-FLAG M2 affinity agarose resin (Sigma) at 4°C overnight, followed by thorough wash in the IP buffer. Washed resin was boiled with 1x SDS gel loading buffer and used in western blotting.

### Protein purification and *in vitro* binding assays

His-tagged mouse *Sox2* was cloned into pET28a (Novagen), and induced by Isopropyl β-D-1-thiogalactopyranoside in BL21 STAR DE3 *E*. *coli* cells (Invitrogen). Cell pellets were lysed in the lysis buffer containing 800mM NaCl, 50mM Tris [pH7.5], 10% Glycerol, 0.5% NP40, 1X EDTA-free protease inhibitor cocktail (Roche), 50mM Imidazole, 0.5mg/ml Lysozyme, and 5mM β-mercaptoethanol. Cell lysate was sonicated and cleared by centrifugation, and then incubated with Ni-NTA resin (GE healthcare). The resin was extensively washed by the lysis buffer and then by the binding buffer (50 mM Tris pH 7.4, 300 mM NaCl, 10% Glycerol, 0.1% NP40, 30mM Imidazole), and was checked for bound protein by SDS-PAGE and coomassie blue staining. Sf9 insect cells (Invitrogen) were infected with baculoviruses expressing F-Ash2l and F-Wdr5 [27] for 72 hours. Cells were lysed in 500mM NaCl, 50mM Tris [pH7.5], 10% Glycerol, 0.1% NP40, protease inhibitor cocktail (Roche), and cleared by centrifugation. The supernatant was incubated with anti-FLAG M2 resin (Sigma) at 4°C for 6 hours and extensively washed with BC500 and 0.5% NP40, followed by BC100 and 0.1% NP40. Bound proteins were eluted with 0.4mg/ml FLAG peptide (Sigma) in BC100 and 0.1%NP40.

For in vitro binding assays, 4μg His-Sox2 on resin was pre-incubated with 100μl binding buffer (above) containing 1% bovine serum albumin (BSA), and further incubated at 4°C overnight after addition of 400ng purified F-Ash2l or F-Wdr5. Resin was extensively washed with the binding buffer (no BSA) and was checked for bound proteins by SDS-PAGE and western blotting.

### Western blotting, RNA extraction, chromatin immunoprecipitation (ChIP), and quantitative PCR (qPCR)

Antibodies were described before [27] or are as follows: anti-HA [12CA5] (Roche, 11583816001, for western); anti-HA (Abcam, ab9110, for ChIP); anti-Myc (Santa Cruz Biotechnology, sc-764x) anti-Oct4 (Santa Cruz Biotechnology, sc-8628x). RNA extraction and ChIP assays were done as described [27]. qPCR was performed with SYBR Advantage qPCR Premix (Clontech) on a ViiA7 Real-Time PCR System (Applied Biosystems). Primers used are listed in Table 1.

**Table 1:** Primers for qPCR.

App.	Species	gene	Forward start	Forward primer (5’-3’)	Backward start	Reverse primer (5’-3’)
RT	mouse	Gapdh		CATCTTCTTGTGCAGTGCCAG		GGCAACAATCTCCACTTTGCC
RT	mouse	Actb		CCTTCAACACCCCAGCCATGTACG		GGCACAGTGTGGGTGACCCCGTC
RT	mouse	Rbbp5		TGGACAGAACTACCCAGAGGA		CCATCGTTACAGCCAACAGC
RT	mouse	Ash2l		TGACACCTTTGGAATAGACACG		AAGGCACTAAGGCACATTTCTT
RT	mouse	Wdr5		GTCTCAGCCGTTCATTTCAACC		CGAAGGACACTGGAGGATTGT
RT	mouse	Dpy30		ACCCTCACTCTGAGTACGGG		GGACTGTAGATCCACCTTCTGT
RT	mouse	Set1a		TGCTGTCCCTCGTAGACTGG		GGCTCTTTCCGTTTTACCTTGA
RT	mouse	Set1b		GTGAAGTCCGGTGAGCACAA		CAGGAGGCGATTCGGTCTTTG
RT	mouse	Mll1		GCAGATTGTAAGACGGCGAG		GAGAGGGGGTGTTCCTTCCTT
RT	mouse	Mll2		GATGAGAATGGCTCGTTGTGG		TCTATCTTGTCACACTTCCGGTA
RT	mouse	Mll3		TCAGTGCCATTCAAGGGTCC		ACCCATGAGTGGATGGTGAGA
RT	mouse	Mll4		GAGGACTCGCTCATGTCCCT		GCGGAGATAGGTGTGGCTC
RT	mouse	Oct4		ACATCGCCAATCAGCTTGG		AGAACCATACTCGAACCACATCC
RT	mouse	Sox2		ACAGATGCAACCGATGCACC		TGGAGTTGTACTGCAGGGCG
RT	mouse	Klf4		GCACACCTGCGAACTCACAC		CCGTCCCAGTCACAGTGGTAA
RT	mouse	Myc		CCACCAGCAGCGACTCTGA		TGCCTCTTCTCCACAGACACC
RT		IRES		AACAGACCTTGCATTCCTTTGGCG		TAAGGCCGGTGTGCGTTTGTCTAT
RT		GFP		TCTTGTAGTTGCCGTCGTCCTTGA		TGACCCTGAAGTTCATCTGCACCA
RT	mouse	Nanog		CCTCCAGCAGATGCAAGAACTC		CTTCAACCACTGGTTTTTCTGCC
ChIP	human	Wdr5 P1	TSS -266	AGGGCACACTAGCACTTTCCTGTA	TSS -187	TTGACTGGTGCAGGCCCTTCTAAT
ChIP	human	Wdr5 P2	TSS +391	ACTGGGTCCTCTTTCCCGCA	TSS +492	ACTGAGTGGCTTGGTGGGTCT
ChIP	human	Wdr5 dst	Chr 9: 134,211,630	AGCTCCATGCCACTGCCTTACTTA	Chr 9: 134,211,824	TGCCTTCTTTCTCCACAGCAGCTA
ChIP	human	Dpy30	TSS -332	TGAGGAAGTTCAGTTGGTCGAGGA	TSS -231	ACCAAGTGACCAGCCCAGTTAGAA
ChIP	mouse	Cad	TSS -388	GCCATGTCGCAGCCAAGAAGATTT	TSS -307	CAATGGCCGCTTCAGCCTTAAACA
ChIP	mouse	Wdr5 P1	TSS -254	ACCAAGAGGTTTCCCAACAGTCCT	TSS -92	ACTGGTGTTCGGTAACTGCAGACT
ChIP	mouse	Wdr5 P2	TSS +373	CACTGGGAGTGGAATTCTCTG	TSS +486	CTAGGACACAAATGAGGGTTGT
ChIP	mouse	Dpy30	TSS -596	TCACAAGCTTGAACCATCACCCAG	TSS -494	TCTGCCTCCCAAGTGCTGGAATTA
ChIP	mouse	Dpy30	TSS +280	ACTGTGAACCCAGAGGTTGTGCTA	TSS +359	AGGTGTATGAGAAGGGTTGAGCCA
ChIP	mouse	Dpy30	TSS +10244	TCTTGGGCAGTAACTGTAGCAGCA		AAGTGCTTGCCAGTACTCAGCTCT
ChIP	mouse	Dpy30	TSS +23114	CCGTGCTTCCCACAAAGCAAATGT		CATGCCACCAGCAACATTAGCACA
ChIP	mouse	Intergenic	Chr8: 72,806,101	AAGGGGCCTCTGCTTAAAAA	Chr8: 72,806,240	AGAGCTCCATGGCAGGTAGA
ChIP	mouse	Fgf4	TSS +681	ACCGGTAGACAGGAGATGAG	TSS +769	ACTCTAAGCCTCTTGGGATCT
ChIP	mouse	Irf6	Ref. (15)	GAGGGAGGACAGACACCTGA		GCCGTCCCAAAACTACTTGA
ChIP	mouse	Cdh1	Ref. (15)	CCGAGCTCAGTGTTTGCTC		CAGGACCCTCCACATACCTG
ChIP	mouse	Sall4	Ref. (61)	AACCTGCATTCTCCTACAGACC		TTTCTTTAATGCCTGCATTTTG
ChIP	mouse	Oct4-A	Ref. (61)	GACGGCAGATGCATAACAAA		AGGAAGGGCTAGGACGAGAG
ChIP	mouse	Oct4-B	Ref. (61)	TGTGAACTTGGCGGCTTC		CCTCCACTCTGTCATGCTCA
ChIP	mouse	Postn	Ref. (15)	TATGCTCTGCTGCTGCTGTT		AACAAGCCAGGGACTTACCC

## Results

### Reprogramming factors promote expression of core subunits of the Set1/Mll complexes

**Fig 1:**
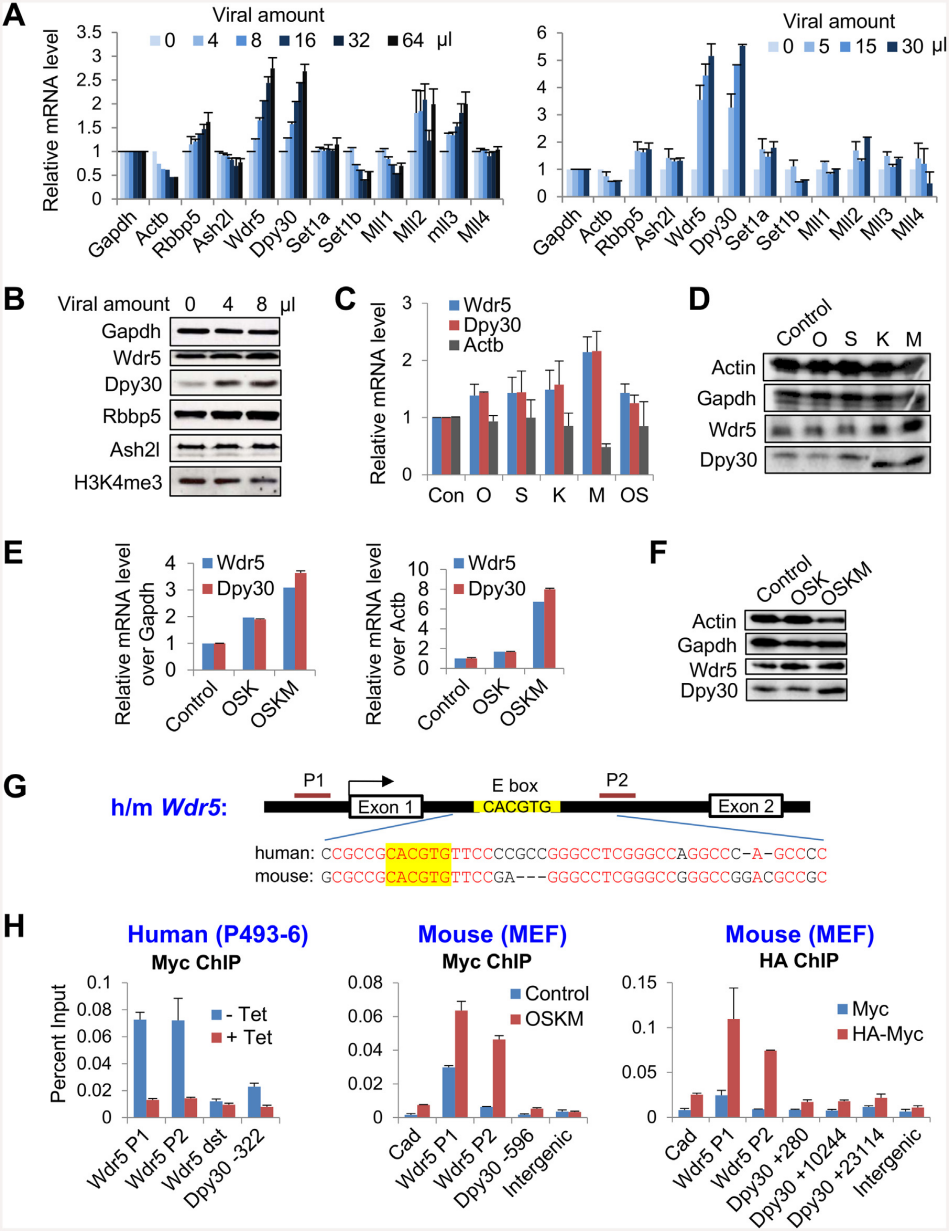
The reprogramming factors promote the expression of the core subunits of Set1/Mll complexes. (A) MEFs were infected with increasing dose of FUW-M2rtTA and TetO-FUW-OSKM viruses in two independent infection experiments, and treated with doxycycline for 2 days. The mRNA levels of Set1/Mll complex subunits was determined by RT-qPCR and normalized by *Gapdh*. Average ± range of values from duplicate assays are plotted. (B) MEFs were infected with increasing dose of FUW-M2rtTA and TetO-FUW-OSKM viruses and treated with doxycycline for 2 days, and the expression of core subunits and global H3K4me3 were examined by western blotting. (C and D) MEFs were infected with virus expressing indicated individual reprogramming factor together with FUW-M2rtTA virus. MEFs infected with only FUW-M2rtTA virus was used as control (Con), and some MEFs were co-infected with TetO-FUW-Oct4 and TetO-FUW-Sox2 viruses (OS), in addition to FUW-M2rtTA. After induction with doxycycline for 2 days, the mRNA (C) and protein (D) levels of indicated Set1/Mll complex subunits were determined by RT-qPCR and normalized by *Gapdh* (C) and western blotting (D). Average ± SD from 3 independent infections are plotted in (C). (E and F) MEFs were infected with viral mixes that expressed individual Oct4, Sox2, Klf4 (OSK) or Oct4, Sox2, Klf4, and c-Myc (OSKM), in addition to FUW-M2rtTA, and MEFs infected with only FUW-M2rtTA virus was used as control. After induction with doxycycline for 2 days, the mRNA levels (E) of indicated core subunits were determined by RT-qPCR and normalized by *Gapdh* (E, left) or *Actb* (E, right). Average ± range of values from 2 independent infections are plotted. The indicated proteins were determined by western blotting (F). (G) Structure of human and mouse *Wdr5* genes showing the highly conserved intronic sequences flanking the canonic E box. Identical residuals between human and mouse are in red. (H) Left: P493-6 cells were cultured in the absence (-Tet, Myc on) or presence of (+Tet, Myc off) tetracycline, and used for Myc ChIP followed by qPCR on indicated loci. Middle: MEFs were infected with only FUW-M2rtTA virus (control) or with FUW-M2rtTA and TetO-FUW-OSKM viruses (OSKM), induced with doxycycline, and used for Myc ChIP followed by qPCR on indicated loci. Right: MEFs were infected with FUW-M2rtTA and TetO-FUW-Myc virus (Myc) or FUW-M2rtTA and TetO-FUW-HA-Myc viruses (HA-Myc), induced with doxycycline, and used for HA ChIP followed by qPCR on indicated loci. Wdr5 dst, a *Wdr5* downstream region. *Cad* is a previously established Myc target [63] and used as a positive control. Note that there are E boxes at +10088 and +23063 bp in the *Dpy30* gene body. Average ± SD from triplicate assays are plotted, except for Myc ChIP in MEFs, for which Average ± range of values from duplicate assays are plotted. The differences between blue and red bars in all three panels are statistically significant (P<0.05 in 2-tailed Student’s *t*-test) for all loci except for the “Wdr5 dst” and the “Intergenic” sites.

To address the initial effects on the machineries responsible for epigenetic priming upon the ectopic expression of the reprogramming factors OSKM, we determined the expression of all of the major integral subunits in the Set1/Mll complexes including the catalytic and core subunits upon expression of increasing doses of OSKM in mouse embryonic fibroblasts (MEFs). The mRNA levels of several subunits, especially Wdr5 and Dpy30, were markedly up-regulated by OSKM in a dose-dependent manner (Fig 1A). We confirmed such up-regulation at the protein level (Fig 1B). Global H3K4 trimethylation (H3K4me3), however, was not enhanced at the initial stage following OSKM expression (Fig 1B), probably due to the modest effect on other subunits (especially the catalytic subunits) of the complexes, which are also required for methylation. These results also suggest that locus-specific dynamics of histone methylation, rather than the change of the global methylation, set the stage for changes of transcriptional programs in the initial stage of reprogramming. This notion is supported by a recent report [17].

We next sought to identify the specific factors among OSKM that promote the expression of *Wdr5* and *Dpy30*. We found that individual expression of O, S, K or M could all up-regulate *Wdr5* and *Dpy30* expression (Fig 1C) at the RNA level. Myc had the strongest effect (Fig 1C), although the fold increase of Myc expression over uninfected MEFs is typically lower than those of the other reprogramming factors (which are usually expressed at low or undetectable levels in uninfected MEFs). The results were confirmed at the protein level (Fig 1D). As Oct4 and Sox2 are known to often act together on target gene transcription [44, 45], we co-infected MEFs with viruses expressing Oct4 and Sox2. Such co-infection, however, did not increase the effect on *Dpy30* and *Wdr5* expression (Fig 1C). We cannot exclude the possibility that cells may have only taken up virus encoding one factor, but this possibility should be low, given that co-infection of MEFs with our high titer viral mix separately encoding O, S, K, and M efficiently reprogrammed the MEFs to pluripotency (data not shown). Therefore, we conclude that although all four factors contribute to the initial up-regulation of the core subunits of H3K4 methyltransferase complexes, Myc plays a major role in such regulation.

As Myc is not essential but stimulatory for induction of iPS cells [46, 47], we sought to compare the effects of the reprogramming factors with and without Myc on the expression of the Set1/Mll complex subunits. Our results showed that, while OSK modestly enhanced both the mRNA and the protein levels of Wdr5 and Dpy30, OSKM strongly increased the expression of these core subunits (Fig 1E and 1F).

To determine if Myc directly regulates *Wdr5* and *Dpy30* expression, we performed ChIP assays for Myc in human P493-6 cells and in MEFs that ectopically express Myc either by OSKM virus or HA-tagged Myc virus. P493-6 is an immortalized B cell line that expresses a tetracycline-repressible *Myc* transgene but negligible endogenous *Myc* [43]. We found that Myc bound to both human and mouse *Wdr5* gene at the promoter and a region in intron 1 close to a canonical E box (5’-CACGTG-3’) surrounded by extraordinarily conserved intronic sequences (Fig 1G and 1H). Similarly, Myc also binds to the promoter region of *Dpy30* gene (Fig 1H). Moreover, after a more thorough examination, we found 6 canonical E boxs at +5451, +10088, +14600, +15385, +19350, +23063 bp in the mouse *Dpy30* gene body (about 24.5 kb in total size), while no E box was found in the 20kb region downstream the gene. Our ChIP assay showed that, in addition to the binding at TSS, HA-Myc also bound to regions near at least two E boxes in the *Dpy30* gene body at +10088 and +23063 bp (Fig 1H, right panel). These results show extensive albeit relatively weak binding of Myc to *Dpy30* gene. Thus, Myc directly promotes the expression of two core subunits of the H3K4 methyltransferase complexes with a potential functional implication.

### Dynamic recruitment of Dpy30 during reprogramming

**Fig 2:**
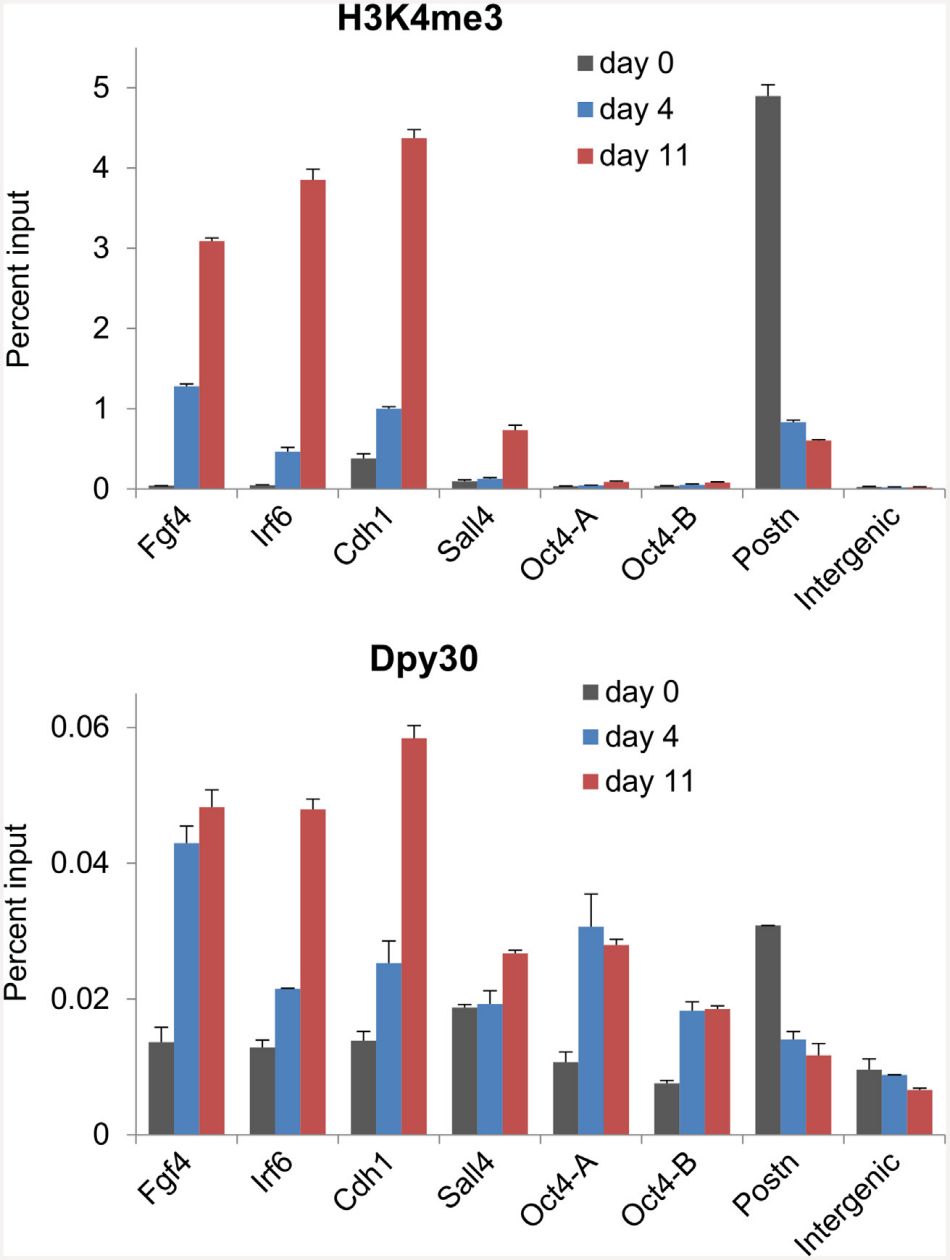
Dynamic recruitment of Dpy30 during cellular reprogramming. MEFs were infected with FUW-M2rtTA and TetO-FUW-OSKM viruses and cultured under the reprogramming condition. At days 0, 4 and 11 during reprogramming, cells were used for H3K4me3 and Dpy30 ChIP followed by qPCR on indicated loci. Oct4-A and Oct4-B are two different primers at the *Oct4* promoter [61]. Average ± range of values from duplicate assays are plotted, and results are representative of 3 independent biological repeats.

The direct promotion of the expression of the Set1/Mll complex core subunits by the reprogramming factors suggests that these subunits may be functionally involved in reprogramming the epigenetic landscape back to that of pluripotency. To understand the molecular mechanisms underlying the dynamics of H3K4 methylation during cellular reprogramming, we sought to determine the binding dynamics of the enzymatic complexes in this process. Consistent with previous reports [15, 17, 18], we found that H3K4me3 was markedly enhanced at promoters of pluripotency-associated genes including *Fgf4*, *Irf4*, *Cdh1*, and *Sall4*, but reduced at *Postn*, a somatic determinant gene, on days 4 and 11 during reprogramming (Fig 2, top). We then found that Dpy30 was increasingly recruited to *Fgf4*, *Irf4*, *Cdh1*, and *Sall4* promoters, while leaving *Postn* in this process (Fig 2, bottom). The changes of local H3K4me3 and Dpy30 binding are in general correlated for most of these loci, but not in a very quantitative manner for all loci (Fig 2). Consistent with findings that *Oct4* promoter does not significantly gain H3K4 methylation until in the final stage [15, 17], our results here also show little H3K4me3 at the *Oct4* promoter on days 4 or 11 during reprogramming (Fig 2, top). Interestingly, Dpy30 binding significantly increased at the *Oct4* promoter (comparable to that at *Irf6* and *Cdh1*) from day 0 to 4 during reprogramming, without eliciting much increase of local H3K4me3 (Fig 2). These results suggest that the reprogramming factors mediate the changes in local histone modifications primarily through affecting the recruitment of the enzymes to those loci, while other mechanisms are also involved, possibly in a gene-dependent manner.

### Interaction of the Set1/Mll complex core subunits with the reprogramming factors

**Fig 3:**
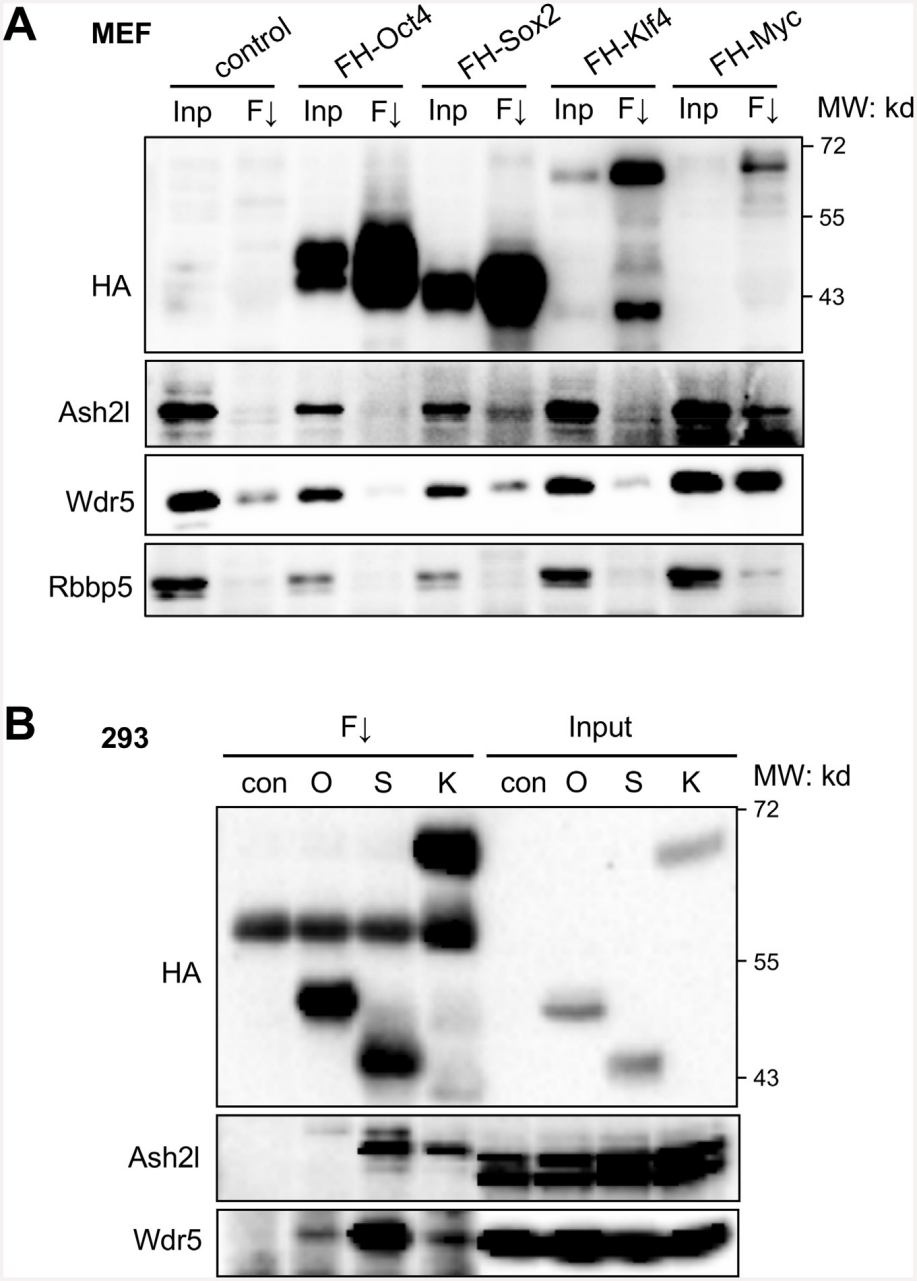
Interaction of the Set1/Mll complex core subunits with the reprogramming factors. (A) MEFs were infected with only FUW-M2rtTA virus (control), or with FUW-M2rtTA and virus expressing the indicated FLAG-HA-tagged individual reprograming factor. After induction with doxycycline for 2 days, co-immunoprecipitation was performed by anti-FLAG M2 resin. Inp, input (4%); F↓, anti-FLAG immunoprecipitation. (B) 293 cells were stably transfected with empty vector (con) or plasmid expressing the indicated FLAG-HA-tagged individual reprograming factor. Co-immunoprecipitation was performed by anti-FLAG M2 resin. Input (2.5%) was loaded.

The dynamic recruitment of Dpy30 following OSKM expression suggests that the Set1/Mll complexes may be recruited to relevant chromatin targets by their physical interaction with the reprogramming factors. We thus characterized such interactions between the core subunits of the Set1/Mll complexes with each of OSKM. We found that, Sox2 and Myc, when individually expressed in MEFs, bind to endogenous Ash2l and Wdr5 (Fig 3A). Possible binding of Oct4 and Klf4 with these subunits was difficult to detect in infected MEFs due to the background signal in control MEFs (Fig 3A). We then performed co-IP in 293 cells expressing individual Oct4, Sox2 or Klf4, and found that they all bound to endogenous Ash2l and Wdr5, yet with varying affinity (Fig 3B). Among OSK, Oct4 was found to be the weakest in binding to the core subunits including Wdr5 and Ash2l, while Sox2 was the strongest in binding to these two subunits of Set1/Mll complexes (Fig 3B).

### The HMG domain of Sox2 mediates its direct binding to Ash2l of Set1/Mll complexes

**Fig 4:**
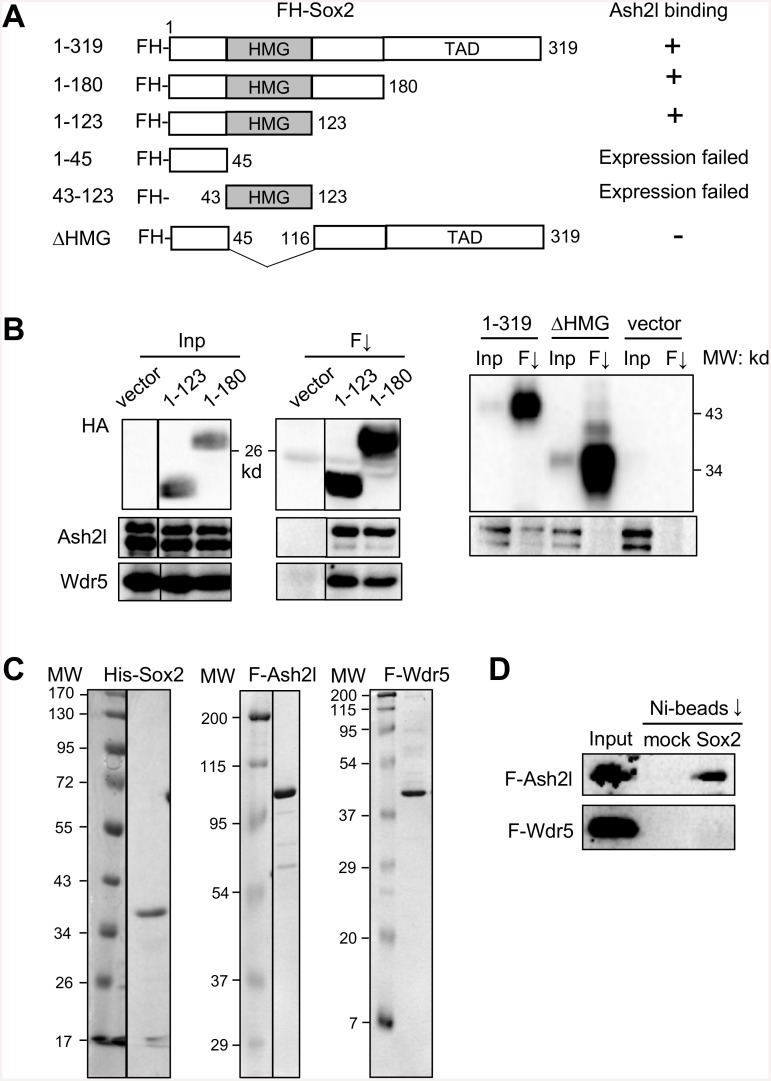
The HMG domain of Sox2 mediates its direct binding to Ash2l of Set1/Mll complexes. (A) Diagram for truncation mutants of FLAG-HA-tagged Sox2 showing their domain structures and whether the protein binds to Ash2l as summarized from (B). (B) 293T cells were transfected with indicated FLAG-HA-tagged Sox2 mutants, and co-IP was performed by anti-FLAG M2 resin. Wdr5 blotting for the right panel is not shown due to a fortuitous cross-reactivity with ΔHMG. TAD: transcriptional activation domain. Inp, input (2.5%); F↓, anti-FLAG immunoprecipitation. (C) Purified proteins were examined by SDS-PAGE along with the molecular weight ladder on the left, and visualized by coomassie blue staining. (D) In vitro binding assay for Ni bead-bound His-Sox2 with FLAG-Ash2l and FLAG-Wdr5, and examined by western blotting using anti-FLAG antibody. Ni-beads that was not used for His-Sox2 purification was used as the mock control in the binding assay. Input (20%) was loaded.

We next determined which domain of Sox2 mediates its binding to Set1/Mll complex core subunits by examining the binding of various truncations of Sox2 with endogenous Ash2l and Wdr5 (Fig 4A). While Sox2 (1–123) containing the HMG domain was sufficient to bind to Ash2l and Wdr5, the Sox2 mutant missing the HMG domain (ΔHMG) failed to bind to these proteins (Fig 4B), indicating that the HMG domain mediates the binding of Sox2 to Ash2l and Wdr5 (and thus to the Set1/Mll complexes).

We then asked if Sox2 directly binds to the Set1/Mll complex core subunits. We recombinantly expressed His-tagged Sox2 in bacteria, FLAG-tagged Ash2l and Wdr5 in baculovirus-infected insect cells, and purified these proteins by affinity chromatography (Fig 4C). Our in vitro binding assays using these purified proteins showed that the Sox2 directly binds to Ash2l, but not to Wdr5, under a stringent binding condition (Fig 4D).

### Set1/Mll complex core subunits are required for efficient reprogramming

**Fig 5:**
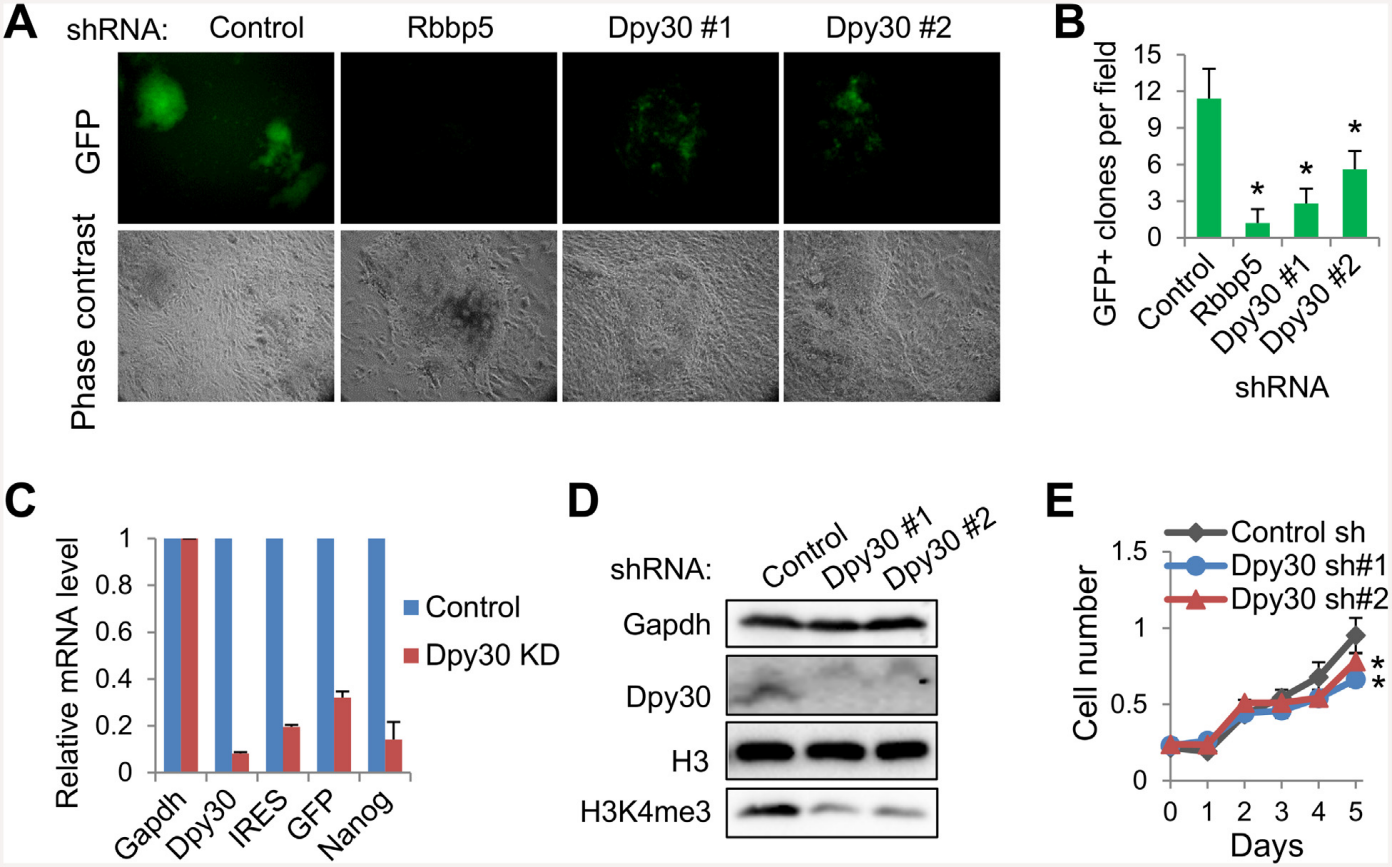
The Set1/Mll complex core subunits are required for efficient reprogramming to pluripotency. (A) Oct4-GFP MEFs stably depleted of Rbbp5 or Dpy30 were used in reprogramming for 16 days, and imaged under microscope. (B) GFP positive clones per field, as averaged ± SD from 10 random fields, were quantified by counting under microscope. Results are representative of over 5 biological repeats. *P<0.05 in 2-tailed Student’s *t*-test between control and each knockdown. (C) The relative mRNA levels of indicated genes were determined by RT-qPCR after reprogramming, and normalized by *Gapdh*. Dpy30 shRNA#1 was used for KD. Results are representative of over 5 biological repeats. (D) Western blotting for MEFs after stable knockdown. (E) Growth curves of the control and Dpy30 KD MEFs as measured by described method [27]. Results are representative of two biological repeats, and average ± SD from triplicate measurements are plotted. *P<0.05 in 2-tailed Student’s *t*-test between control and each knockdown.

Having established the physical interactions between the reprogramming factors with the Set1/Mll complex core subunits, we asked whether these subunits are functionally important in helping fulfill the mission of the reprogramming factors. Through the doxycycline-inducible expression of OSKM in a single polycistronic viral vector [40], we could achieve efficient reprogramming of primary Oct4-GFP MEFs, in which *GFP* is placed downstream of the endogenous *Oct4* locus through an internal ribosome entry site (IRES) and its expression serves as a faithful indicator for the acquisition of pluripotency [48]. We then stably depleted RbbP5 or Dpy30 (by two different shRNAs) in these MEFs, given a crucial role of Rbbp5 in the structural organization of the Set1/Mll complexes [23]. The knockdown (KD) did not affect the formation of dense colonies at the late stage after OSKM expression (Fig 5A, phase contrast), but significantly impaired the GFP induction both qualitatively and quantitatively (Fig 5A and 5B), and reduced the induction of the endogenous *Oct4* (indicated by PCR on *IRES* and *GFP* sequences to distinguish from the viral *Oct4*) and *Nanog* (Fig 5C). We titrated the OSKM viral amounts so that the four factors were expressed at comparable levels in the control versus KD MEFs (data not shown). Consistent with the role of Dpy30 in facilitating H3K4 methylation, global H3K4me3 was markedly reduced in MEFs upon Dpy30 KD (Fig 5D). Cell growth rate was mildly affected by Dpy30 KD in MEFs (Fig 5E). These results indicate that Rbbp5 and Dpy30 in Set1/Mll complexes are important for efficient reprogramming to pluripotency.

### Dpy30 is required for efficient recruitment of Oct4 to its genomic targets

**Fig 6:**
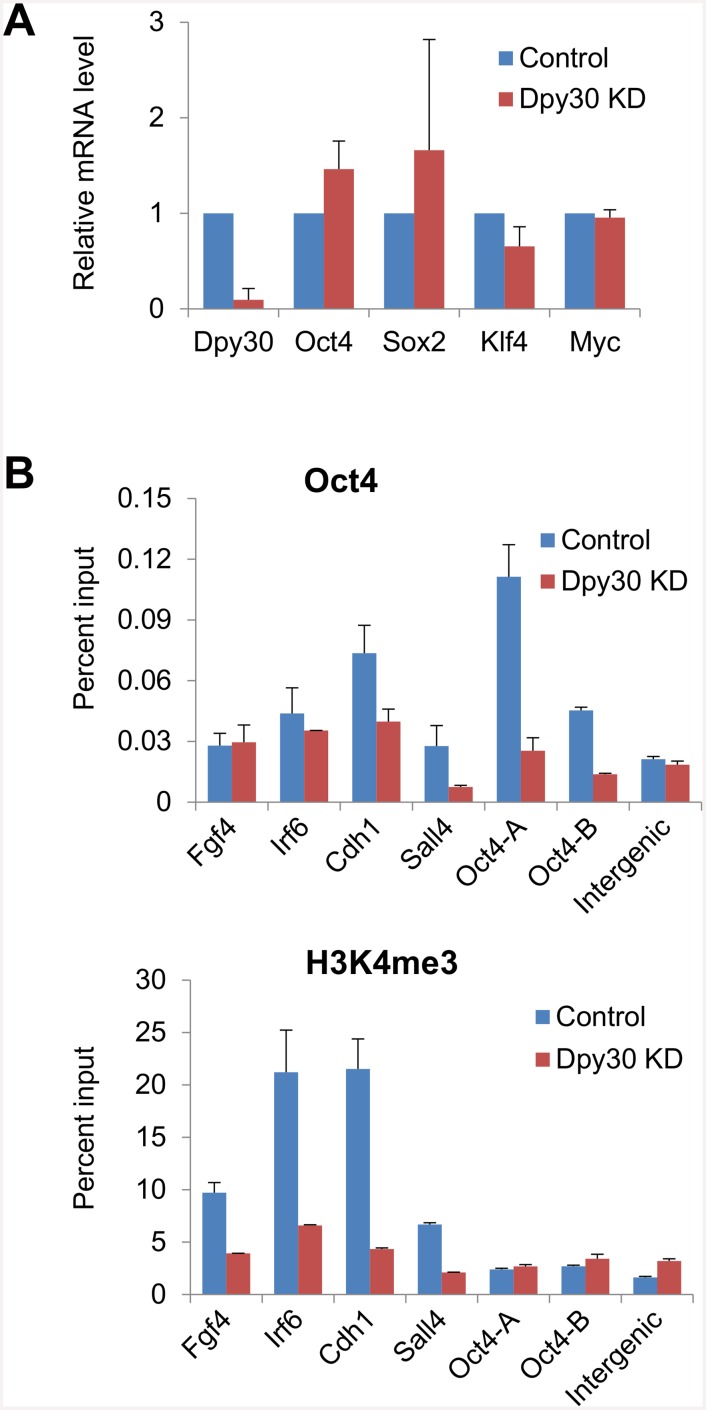
Dpy30 is required for efficient recruitment of Oct4 to its genomic targets. MEFs infected with control shRNA or Dpy30 shRNA#1 (KD) were used in reprogramming for 7 days. (A) Expression of *Dpy30* and *OSKM* was determined by RT-qPCR and normalized by *Gapdh*. Average ± SD from 3 independent infection and reprogramming assays are plotted. (B) ChIP assays for Oct4 and H3K4me3 were performed followed by qPCR on indicated gene promoters or an intergenic site. Average ± range of values from duplicate assays are plotted, and results are representative of 3 independent infection and reprogramming assays.

As reprogramming is driven by the binding of the reprogramming factors to their chromatin target sites and the following execution of the transcriptional programs, we hypothesized that the Set1/Mll complex activity is required for the efficient binding of the reprogramming factors to the chromatin targets. Given the key role of Oct4 in a successful reprogramming to pluripotency, we focused on the genomic binding of Oct4. We performed OSKM-mediated reprogramming on the control and stable Dpy30 KD MEFs with titrated OSKM viral amounts so that the reprogramming factors were expressed at the comparable levels in the Dpy30 KD versus control cells (Fig 6A). In support of our hypothesis, Dpy30 KD markedly reduced the recruitment of Oct4 to several pluripotency gene promoters including the endogenous *Oct4* promoter 7 days after OSKM induction (Fig 6B, top). In an attempt to study molecular mechanisms underlying the regulation of Oct4 recruitment by Dpy30, we examined the effect of Dpy30 KD on H3K4 methylation at relevant gene promoters. We found that Dpy30 KD greatly reduced H3K4me3 at the promoters of *Fgf4*, *Irf6*, *Cdh1*, and *Sall4* (Fig 6B, bottom). Minimal H3K4me3, however, was at the *Oct4* promoter at this stage of reprogramming, and consequently, Dpy30 KD had little effect on H3K4me3 at these promoters (Fig 6B, bottom). These results provide a mechanistic basis for the importance of Dpy30 (and potentially other core subunits of the Set1/Mll complexes) in cellular reprogramming.

## Discussion

**Fig 7:**
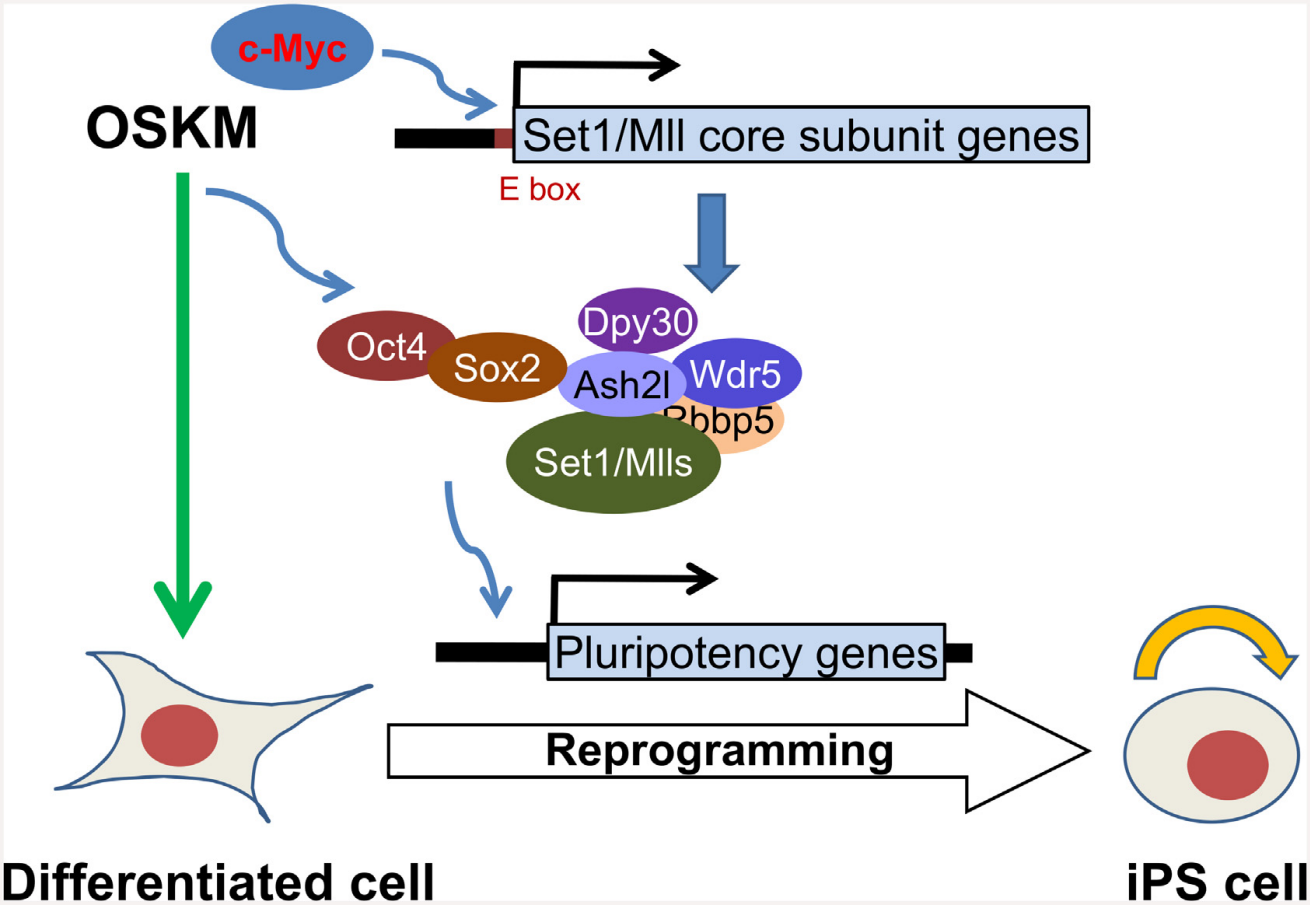
A model for the role of the core subunits of Set1/Mll complexes in cellular reprogramming. This model depicts two different levels of mechanisms by which the reprogramming factors coordinate with the core subunits of Set1/Mll complexes to facilitate cellular reprogramming of differentiated cells to pluripotency. See Discussion for details.

Cellular reprogramming is essentially an epigenetic process, and an efficient reprogramming probably requires a close coordination between the initiating transcription factors and the responsive epigenetic factors. Our work here outlines a model (Fig 7) for such coordination between OSKM and the core subunits of Set1/Mll complexes, the major H3K4 methyltransferases in mammalian cells. OSKM, especially Myc, stimulates the expression of the core subunits, most prominently Dpy30 and Wdr5. Sox2 also directly interacts with Ash2l and likely guides the localization of the Set1/Mll complexes to the promoters of pluripotency genes. These complexes remodels the local chromatin to help the further recruitment of reprogramming factors including Oct4. These coordinated actions of the transcription factors and chromatin modifiers eventually lead to the change of the cell fate to pluripotency.

Our findings that OSKM can bind to the Set1/Mll complex core subunits combined with the increased recruitment of these subunits to genomic sites following OSKM induction suggest a direct role of the reprogramming factors in recruiting the H3K4 methyltransferases to epigenetically prime the transcription programs for reprogramming. The increase of Dpy30 binding and H3K4me3 at the pluripotency-associated genes is unlikely a mere result of passive spread of overall increased levels of Dpy30 and histone methylation, because (i) the reduced Dpy30 binding and H3K4me3 at *Postn* (Fig 2) following OSKM expression suggests a regulated and locus-specific dynamics of methyltransferase binding, and (ii) the increase of the Dpy30 recruitment to several promoters (*e*.*g*. *Irf6*, *Cdh1*, and *Sall4*) occurs at the later stage of the reprogramming (Fig 2), while the overall upregulation of Dpy30 occurs shortly after OSKM expression. The reduced Dpy30 binding and H3K4me3 at *Postn* and presumably other loci that get silenced during reprogramming cannot be explained by a direct recruitment of Set1/Mll core subunits by OSKM to those sites, and is most likely an indirect result initiated by the reprogramming factors. The recruitment of Dpy30 (and presumably the H3K4 methyltransferase complexes) at the *Oct4* promoter is insufficient to enhance H3K4me3 at the same site in the intermediate stage of reprogramming. Our results suggest that, while regulated recruitment of the epigenetic machinery is the major determinant, multiple mechanisms are responsible for the dynamic epigenetic regulation involved in reprogramming.

Our findings are consistent with previous reports on the direct interaction of Myc with Ash2l [49] and that of Oct4 with Wdr5 [28]. They are also consistent with the broad association of promoters gaining H3K4me2 and targets for OSKM, predominantly Oct4 and Sox2 [15]. Since Sox2 binds to the core subunits of Set1/Mll complexes much more strongly than Oct4, it is likely that Sox2 may assist its close partner Oct4 to bring the H3K4 methyltransferases to their common targets. The interaction of the Set1/Mll complexes with key cell fate-regulatory transcription factors appears to be highly conserved in evolution, as a Wdr5 ortholog in *C*. *elegans* was recently shown to interact with Sox2 and CEH-6 (OCT) [50].

Although Myc regulates a large number of expressed genes in the genome [51–54], it is unlikely that its regulation of all of the targets is critical. The phylogenetic conservation of the Myc binding site within intron 1 of *Wdr5* is extraordinary for an intronic sequence, suggesting that this regulation is likely to be physiologically important. Our results may partially explain the previously finding that *Myc*-null mouse embryos have greatly reduced bulk level of H3K4me3 [55]. The up-regulation of *Wdr5* and *Dpy30* upon OSKM (especially Myc) expression is not sufficient to enhance global H3K4me3, but it is likely to facilitate alteration of gene-specific methylation, or prepare for the much elevated methylation at the later stage of reprogramming. As the Myc regulatory network is pervasively involved in the regulation of cell growth, metabolism, and tumorigenesis [56], Set1/Mll complexes are unlikely to be specifically involved in regulating maintenance or acquisition of pluripotency. In this sense, depletion of many subunits in the Set1/Mll complexes commonly affects cell proliferation in a cell context-dependent manner [28, 29, 57]. It is possible that the altered cell proliferation or other potential cellular effects upon Dpy30 KD may affect the reprogramming efficiency. However, the effect of cell proliferation on reprogramming is not definitive or unidirectional since both positive and negative effects of cell proliferation on reprogramming have been reported [58, 59]. Furthermore, dense colonies still form in Rbbp5 or Dpy30 KD cells during reprogramming, except that these colonies show no or little GFP signals (Fig 5A). Finally, the impaired target binding of Oct4 upon Dpy30 KD suggests an important role of Dpy30 for the molecular activity of the reprogramming factors through regulating the chromatin accessibility.

The molecular basis for the requirement of Set1/Mll complex subunits for efficient cellular reprogramming is not well understood, although H3K4 methylation is generally thought to be the major mediator. It is proposed that an efficient engagement of Oct4, Sox2 and Klf4 at the pluripotency-associated genes, especially at the intermediate stage of reprogramming, is a major barrier to the completion of reprogramming [60, 61]. Our results show that Dpy30 is required for the efficient binding of the exogenous Oct4 to its genomic targets including several pluripotency-associated gene promoters at the intermediate stage of reprogramming, providing a molecular basis for Dpy30 (and potentially other core subunits) to regulate reprogramming. Our analysis of H3K4me3 at the same promoters, however, reveals a lack of good correlation of the effect on H3K4me3 with that on Oct4 recruitment. While H3K4me3 is markedly reduced at the *Irf6*, *Cdh1*, and *Sall4* promoters in Dpy30 KD cells, it is not affected at the *Oct4* promoter, which lacks H3K4me3 induction in control cells at this stage of reprogramming. Oct4 recruitment to these promoters, however, is still critically dependent on Dpy30, regardless of the locus-specific status of H3K4me3. Indeed, Oct4, Sox2 and Klf4 are known to extensively access closed chromatin loci without active histone marks at the early stage of reprogramming [62]. Therefore, although H3K4me3 facilitated by Dpy30 at many pluripotency-associated promoters may functionally contribute to efficient Oct4 recruitment to those sites at certain stage of reprogramming, our results on *Oct4* promoter suggest the existence of an alternative or complementary mechanism by which Dpy30 facilitates the recruitment of a reprogramming factor. We note that this alternative mechanism does not necessarily exclude the involvement of the H3K4 methylation activity of the enzymatic complexes that may affect global chromatin environment and thus secondarily affect the local genomic accessibility (*i*.*e*., indirect effect for a particular locus).

Based on this and our previous work [27], we conclude that certain core subunits of the Set1/Mll complexes including Rbbp5 and Dpy30 are important for the plasticity of cell identity in the two-way transitions between the pluripotent and differentiated states. Loss of Rbbp5 or Dpy30 results in a rigid transcriptional program and a fixed cell identity. Our findings have added functional significance and mechanistic basis for the epigenetic priming of genes for reprogramming, and further demonstrate a profound impact of the epigenetic machinery on the plasticity of cell fate. This conclusion is also in line with a recent report that demonstrates an important role of the H3K4 methylation activity in ensuring robust transdifferentiation of hindgut cells into motor neurons in *C*. *elegans* [50].
